# Necroptotic Cell Death Signaling and Execution Pathway: Lessons from Knockout Mice

**DOI:** 10.1155/2015/128076

**Published:** 2015-09-27

**Authors:** José Belizário, Luiz Vieira-Cordeiro, Sylvia Enns

**Affiliations:** ^1^Department of Pharmacology, Institute of Biomedical Sciences, University of São Paulo, 05508-900 São Paulo, SP, Brazil; ^2^Department of Animal Science, Federal Rural University of the Semiarid Region, 59625-900 Mossoró, RN, Brazil

## Abstract

Under stress conditions, cells in living tissue die by apoptosis or necrosis depending on the activation of the key molecules within a dying cell that either transduce cell survival or death signals that actively destroy the sentenced cell. Multiple extracellular (pH, heat, oxidants, and detergents) or intracellular (DNA damage and Ca^2+^ overload) stress conditions trigger various types of the nuclear, endoplasmic reticulum (ER), cytoplasmatic, and mitochondrion-centered signaling events that allow cells to preserve the DNA integrity, protein folding, energetic, ionic and redox homeostasis, thus escaping from injury. Along the transition from reversible to irreversible injury, death signaling is highly heterogeneous and damaged cells may engage autophagy, apoptotic, or necrotic cell death programs. Studies on multiple double- and triple- knockout mice identified *caspase-8*, *flip*, and *fadd* genes as key regulators of embryonic lethality and inflammation. Caspase-8 has a critical role in pro- and antinecrotic signaling pathways leading to the activation of receptor interacting protein kinase 1 (RIPK1), RIPK3, and the mixed kinase domain-like (MLKL) for a convergent execution pathway of necroptosis or regulated necrosis. Here we outline the recent discoveries into how the necrotic cell death execution pathway is engaged in many physiological and pathological outcome based on genetic analysis of knockout mice.

## 1. Introduction

Cell death is a crucial process in ontogeny, homeostasis, and pathologies [1, 2]. Over 100 billion cells die in our bodies by different cell death pathways every day. The cells die by apoptosis, a physiological and regulated cell death process which is tolerogenic and partially inflammatory, or necroptosis, a pathological and regulated cell death process, which is inherently immunogenic and elicits intense inflammatory reaction [3, 4]. Pyroptosis [5], immunogenic cell death [6, 7], and other distinct cell death processes have been defined at morphological and biochemical levels [3, 4, 8, 9]. Many questions concerning the cross talk among the cell death regulators, their intracellular signaling pathways, and the immunological consequences remain unanswered. Genetic dissection in simple model organisms [10] and mice models [11] has provided us with critical genes of cell-death pathways that control early and late biochemical and morphological events in organ development and cellular homeostasis. A variety of cell death modalities share extrinsic and intrinsic pathways that integrate mitochondrial metabolism, cell proliferation checkpoints, and DNA repair mechanisms [3, 4, 9]. It is now becoming evident that perturbations of intracellular ionic homeostasis induced by certain transmembrane non- and voltage dependent-channels and ion-linked channel receptors play critical roles in the course of cell death processes [1–5, 9]. Here we will summarize common features of necrosis, apoptosis, and necroptosis and the multiple intracellular signal pathways that regulate their cellular triggering in many physiological and pathological situations. In the end, we will outline and discuss important phenotypes of knockout mice models that serve to define the role of* caspase-8*,* flip*, and* fadd* genes and other major components of apoptotic and necroptotic downstream signaling effectors.

## 2. Cell Death Modalities

### 2.1. Accidental Necrosis

**Figure 1:**
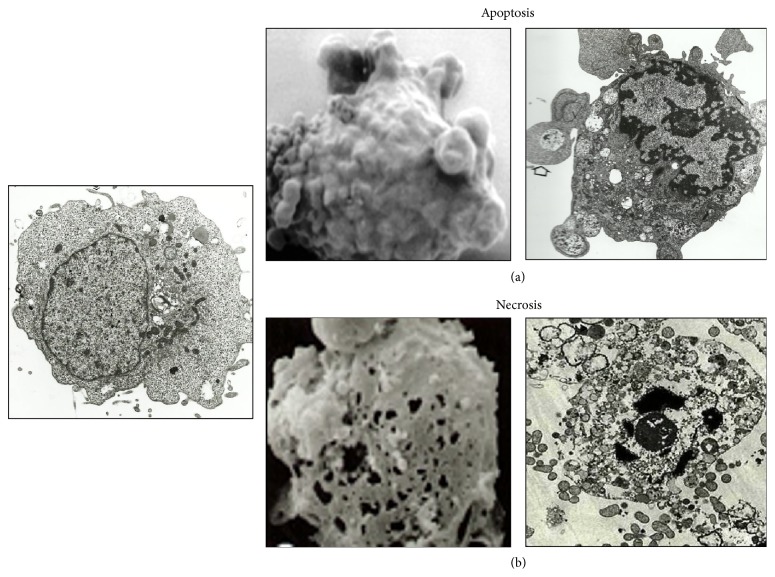
Distinct morphological features of apoptosis and necroptosis. (a) Apoptosis is characterized by cell shrinkage, membrane blebbing condensation, margination of nuclear chromatin, and packaging of apoptotic bodies and its engulfment by neighbor cells. (b) Necroptosis is characterized by the increase in cell volume, swelling of organelles, perforation of plasma membrane, cellular collapse, and release of cellular contents.

Necrosis derives from the Greek word “necros” and has long been used by pathologists to describe morphologically the death of cells or tissue as result of pathological infection, cellular injury, and noxious stimuli [1, 4]. The term necrosis is now referred to as accidental cell death, which is a form of nonregulated, nonspecific, and uncontrolled cell death by meaning of genetic and biochemical interventions [4]. Necrotic death occurs quickly as a consequence of extreme physicochemical stress, such as heat, acidification, osmotic shock, mechanical stress, and freeze-thawing of cells [2]. Necrotic cells are characterized by loss of plasma membrane integrity, increase in cell volume (also known as oncosis), organelle swelling, lack of internucleosomal DNA fragmentation, and cellular collapse (Figure 1). These events occur at early or late stages of cellular collapse due to cellular energy depletion (ATP), mitochondrial permeability transition, increases in cytosolic calcium concentration, high production of free radicals, reactive (activated) oxygen species (ROS), oxidization of membrane lipids, plasma membrane damage and permeability changes, and critical DNA and protein structural damage [2, 8].

### 2.2. Apoptosis

The term “apoptosis” was derived from a Greek word that means “falling off” and was first coined by Kerr et al. [1] to describe a morphological manifestation of cellular demise. The complex cellular morphology known as apoptosis can be confidently recognized by a series of morphological changes at the microscopy level (Figure 1). Apoptosis is characterized by cell shrinkage, membrane blebbing, condensation and margination of nuclear chromatin, degradation of DNA into nucleosomal units (200 bp), and formation of apoptotic bodies. However, the hallmark of an apoptotic process is its dependence on caspase activation [1, 8, 12].

Cells undergo apoptosis in response to extrinsic or intrinsic pathways that are regulated by various antiapoptotic and proapoptotic proteins [8, 13]. The extrinsic pathway is mediated by binding of the tumor necrosis factor (TNF) to its receptor (TNFR-1) which is followed the formation of two TNFR complexes [14]. TNFR-1 complex I comprises the adaptor protein TNFR1-associated death domain protein (TRADD), the death domain- (DD-) containing protein kinase receptor-interacting protein 1 (RIPK1), the ubiquitin E3 ligases TNFR-associated factor 2 (TRAF2), and cellular inhibitor of apoptosis protein 1 (cIAP1). TNFR-1 complex II comprises the adaptor FAS-associated death domain protein (FADD), caspase-8, RIPK1 (IIa), and/or RIPK3 (IIb). E3 ubiquitin ligases, including cIAP-1 and -2 and deubiquitinases, including CYLD (cylindromatosis), A20, Cezanne, HOIL-1/HOIP/Sharpin (LUBAC ubiquitin ligase complex), modify the balance between TNFR-1 complex I and complex II [13–15] leading to either cell survival via activation of nuclear factor-*κ*B (NF-*κ*B) or alternative cell death signaling pathways.

Homodimerization and activation of caspase-8 within either TNFR complex I or II propagate the activation of effector caspases-3, -6, and -7, which then cause cellular destruction by apoptosis without mitochondria participation (known as type I intrinsic pathway). Both caspase-8 and cFLIP (FADD-like IL-1*β*-converting enzyme-inhibitory protein) possess two DED (death effector domain) domains. Their heterodimerization will block the apoptotic signaling cascade, leading to cellular survival and NF-*κ*B-mediated proinflammatory response. Thus the ratio of different c-FLIP isoforms (long and short isoforms) bound to procaspase-8 is a critical regulator of both procaspase-8 dimerization/activation and cell death by apoptosis and necroptosis [16].

The intrinsic pathway is also called the mitochondrial pathway and is absolutely dependent on BAX and BAK protein activation by BH3-only molecules (BH3s) of The BCL-2 (B-cell lymphoma/leukemia 2) family proteins. Various BH3s proteins of the pro-apoptotic family, including BAD, BIK, BID, BIM, BMF, NOXA, and PUMA participate in this process. These proteins interact and dislocate the BH3s proteins of the anti-apoptotic family, including BCL-2, BCL-XL and MCL-1, allowing conformational changes and association of BAX/BAK proteins at specific sites of the mitochondrial outer membrane. Cytochrome *c* is an abundant protein of the mitochondrial inner membrane and acts as an electron transport intermediate. BAX and BAK promote apoptosis by perturbing the permeability of the mitochondrial outer membrane, referred to as MOMP, which lead to the release of cytochrome *c* [17]. The cytochrome *c* released into the cytoplasm stimulates the formation of the apoptosome, a scaffold for activation of caspase-9, which, in turn, cleaves and activates the effector caspases-3, -6, and -7. Mitochondrial membrane permeabilization leads to the release of other apoptogenic proteins, including apoptosis inducing factor (AIF), Smac/Diablo, HtrA2/Omi (serine protease), and endonuclease G, which execute distinct functions in the downstream apoptosis-signaling pathway. Accordingly, new small-molecule inhibitors of Bcl-2 proteins, including ABT-199 and ABT-263, are viewed as promising new anticancer agents.

*In vivo,* apoptotic cells maintain their plasma membrane integrity. However, apoptotic cells under certain conditions promote the exposure or release of ecto-calreticulin, phosphatidylserine, HSP70, HSP90, opsonins, thrombospondin, and high mobility group box 1 (HMGB1), which are known as cDAMPs [18]. The uptake of apoptotic cells by macrophages promotes the release proinflammatory cytokines [19, 20] and cell growth and wound healing through the release of vascular endothelial growth factor (VEGF) and transforming growth factor-*β* (TGF-*β*), respectively [21]. Several cytokines and lipid mediators produced by immune cells and surrounding tissue are involved in the resolution of acute and chronic inflammation [22].

### 2.3. Necroptosis

Necroptosis is referred to as cell death initiated by TNF receptors following chemical suppression of caspases [3]. Peter Vandenabeele and Junying Yuan were the first authors to describe the morphological and biochemical features of necroptosis (more details in Section 4). It is morphologically characterized by the increase in cell volume, swelling of organelles, plasma membrane permeabilization, cellular collapse, and release of cellular contents. Necroptotic cells promote highly inflammatory response as consequence of the release of cytokines, cDAMPs, and PAMPs [18, 20]. Necroptosis is now considered a regulated cell death program that ultimately relays in a core execution pathway to promote final cellular demise [4, 9]. A detailed mechanism for necroptotic cell death execution pathway has been described that involves the integration of many downstream signaling pathways with a trio formed by the receptor interacting protein kinase 1 (RIPK1), RIPK3, and MLKL [23, 24]. Importantly, many cell death triggers can induce necroptosis at various pathological scenarios [9].

**Figure 2:**
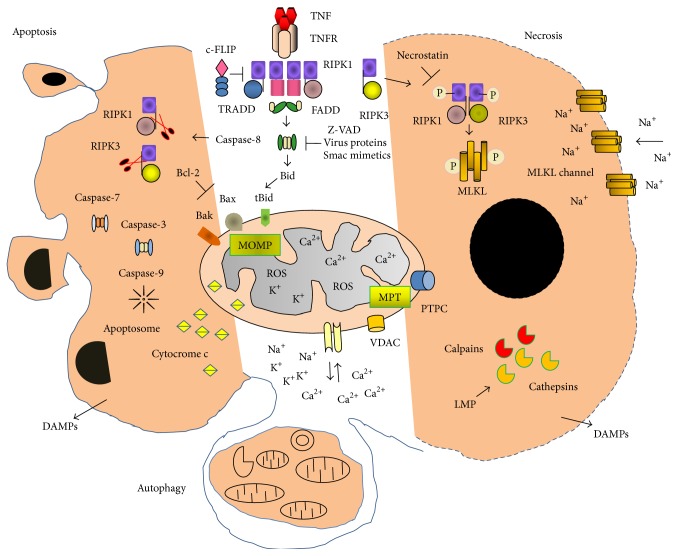
Schematic overview of the multiple signaling pathways to apoptosis, necroptosis and autophagy. TNF-*α* binding to TNFR causes the assembly of a membrane-proximal supramolecular complex including (but not limited to) TRADD, FADD, and RIPK1 (receptor interacting protein kinase 1). Recruitment and activation of caspase-8 play a crucial role in initiation of apoptotic or necrotic cell death. Active caspase-8 cleaves Bid, generating tBid, with together with Bax and Bak promote the mitochondria outer membrane permeabilization (MOMP) allowing the release of cytochrome *c*. Cleavage of both RIP1 and RIP3 by caspase-8 leads to apoptosis, whereas phosphorylation of RIP1 and RIP3 protein kinases causes their activation and in turn the recruitment of MLKL (mixed lineage kinase domain-like). MLKL is phosphorylated by RIP3 and initiated structural changes that led to its insertion in the plasma membrane and formation channels. MLKL channels increase Na^+^ influx, osmotic pressure, and membrane rupture, ending with cell death by necroptosis. Membrane rupture promotes the release of cellular contents and, in particular, various endogenous DAMPs. Various chemotherapeutical drugs, chemical and biological stressors, cause mitochondrial dysfunctions and consequently increase the level of ROS (reactive oxygen species, ROS) generation and collapse of electrochemical gradient, which compromise the ADP/ATP exchange transporter. High Ca^2+^ upload in the matrix favors the transient or irreversible opening or closure of the outer/inner mitochondrial permeability transition pore complex (MPTPC) that is well known to participate in the mitochondrial permeability transition (MPT). This is accompanied by mitochondrial depolarization, loss of membrane potential (ΔΨm), and massive swelling due to influx of ions and water into the matrix. Depending on the extension of cell injury, the cells undergo apoptosis, necrosis, or autophagy programs. Autophagy of damaged organelles constitutes a survival response that prevents cell death. VDAC: the voltage-dependent anion channel, also known as porin; DAMPs: damaged associated-molecular patterns; TNF*α*: tumor necrosis factor *α*; TNFR: tumor necrosis factor receptor; FADD: Fas-associated death domain protein; Z-VAD.fmk: Z-Val-Ala-Asp(OMe)-fluoromethylketone; LMP: lysosomal membrane permeabilization; PTPC: permeability transition pore complex; Smac: second mitochondria-derived activator of caspase.

Recently it has been demonstrated that cells undergo regulated necroptosis after activation canonical and noncanonical extrinsic or intrinsic pathways triggered by TNF-*α*, TLR (toll-like receptors) and NLR (NOD-like receptors) agonists, interferons, viral and bacterial products, and diverse pathophysiological signals [9]. The emerging connections between necroptosis and apoptosis intrinsic and extrinsic pathways are depicted on Figure 2 described in more details in the next sections.

## 3. Mitochondrial Channels and Pores That Control Apoptosis and Necroptosis Cell Death

The role of mitochondria is well defined in apoptotic cell death and accidental necrosis. The mitochondrial involvement in necroptosis is still very preliminary and debatable. Given that various transmembrane non- and voltage dependent-channels and ion-linked channel receptors play critical roles in the course of cell death processes, here we will update on some of the putative mechanisms that contribute to apoptotic and necrotic cell death.

### 3.1. Regulation of Mitochondrial Osmotic Balance

Mitochondria function in a cytosolic milieu containing Na^+^, K^+^, and Ca^2+^ as well as other anions and cations [25]. The inner mitochondrial membrane, however, is impermeable to these ions and their flux and concentrations in the mitochondrial matrix are controlled by different subtypes of pores, channels, and exchangers. The mitochondrial K^+^ balance is controlled by ATP-dependent (KATP) and Ca^2+^-dependent (KCa) K^+^ channels responsible for influx and by K^+^/H^+^ exchanger responsible for removal of excess of matrix K^+^ [26, 27]. Sodium balance is governed by Na^+^/Ca^2+^ (influx) and Na^+^/H^+^ (efflux) exchangers and calcium balance by Ca^2+^ channel (influx) and Na^+^/Ca^2+^ exchangers (efflux). The balance of the main cytoplasmic anions, phosphate and chloride is regulated by numerous carriers and channels [25, 27]. Cell size decreases due to water loss from increased K^+^ and Cl^−^ ionic effluxes. The mitochondrial inner membrane also has unique water channels named aquaporins, which facilitate water or other small-uncharged molecule transport between the cytoplasm and matrix [28]. The rate of water flux in or out of the mitochondrion is determined not only by the osmotic gradient that acts as the driving force for water transport but also by the water permeability of the inner membrane [28].

The mechanisms by which Ca^2+^ ions modulate both oxidative phosphorylation and mitochondrial matrix swelling have been a matter of great debate for decades. Halestrap and colleague [29] demonstrated that an increase in intramitochondrial Ca^2+^ concentration can increase the flux of K^+^ into mitochondria. Since then, several mechanisms have been proposed to explain this finding. First, it was proposed that elevated matrix Ca^2+^ could open a putative mitochondrial large conductance Ca^2+^-activated K^+^ channel (KCa channel) [26, 30]. Activation of calcium efflux pathways via Na^+^/Ca^+^ and Na^+^/H^+^ exchangers (mitochondrial NHE1) during intramitochondrial Ca^2+^ overload causes dissipation of the mitochondrial membrane potential and loss of proton gradient [27]. Loss of proton gradient suppresses the activity of K^+^/H^+^ exchange leading to the mitochondrial swelling. Finally, if the Na^+^/Ca^2+^ exchanger becomes saturated, a high intramitochondrial Ca^2+^ could rise to the level sufficient to facilitate entry of potassium as well as other ions and solutes and inducing massive swelling of the matrix [26]. An increase of Ca^2+^ concentration up to the micromolar level inhibits pyrophosphatase activity in the matrix, which, in turn, may transiently displace adenine nucleotides from adenine nucleotide translocase (ANT) and convert the latter into a potassium channel. Most likely the total net flux of ions is directed toward the mitochondrial matrix and this ion movement, accompanied by osmotically mediated entrance of water, which leads finally to mitochondrial swelling [31]. The swelling could lead to a rupture of the mitochondrial outer membrane allowing further expansion of the matrix. Thus, rupture of the mitochondrial outer membrane has been proposed as alternative way of permeating the release of cytochrome *c* in the apoptotic process, but this is debatable [32].

### 3.2. ROS-Induced Mitochondrial Permeability

ROS (reactive oxygen species) overproduction can lead to severe mitochondrial dysfunction commonly observed in the necrotic cell death process [2]. Reactive oxygen species induce mitochondrial structural oxidation and opening of the permeability transition pores, channels, and exchangers [33]. Under normal metabolic conditions, electron-transporting complexes I, II, III, and IV plus a nonredox H^+^-translocating complex, ATP synthase (also called complex V, FoF1-ATP synthase), coenzyme Q, and cytochrome *c* carry out oxidative phosphorylation [34]. The a- and b-type cytochromes are inaccessible components of large complexes, but cytochrome *c* is monomeric, freely diffusible in the inner membrane, and in equilibrium between the inner membrane, intermembrane space and cristae. A small percentage of the total O_2_ consumed by the mitochondrial electron transport chain in healthy tissues becomes ROS, such as superoxide (O_2_^·−^), hydrogen peroxide (H_2_O_2_), and hydroxyl radical (OH−) [33]. This ROS production occurs primarily in complex I (NADH dehydrogenase) and complex III (ubiquinone-cytochrome *c* reductase). O_2_ itself is also a free radical because it has two unpaired electrons in its outer orbit that make it reactive. Increase in the level of products of the one-electron reduction of O_2_ is known to induce the mitochondrial permeabilization transition. The hypothesis is that pore formation is involved in the organization of a defense system preventing ROS formation. It is proposed that an ROS-induced nonspecific pore opening lowers ROS production due to (a) maximal stimulation of mitochondrial O_2_ consumption and, hence, intracellular lowering of ROS and (b) complete dissipation of mitochondrial membrane potentials and, as a consequence, maximal oxidation of such respiratory chain carriers such as coenzyme Q, which serve as one-electron O_2_ reductant [34].

Earlier studies have shown that OH^·^ oxidize thiol (–SH) groups of sensor proteins which directly promote the activation or opening of the mitochondrial permeability transition pore [33, 34]. The mitochondrial permeability transition pore complex (PTPC) is a nonspecific pore in the inner mitochondrial membrane (IMM) whose opening is triggered by high concentration Ca^2+^ in the matrix [35–37]. Ca^2+^ loading increases the production of ROS, including superoxide. PTP is considered to function as a point of no return for both apoptosis and necrosis. This supramolecular complex may contain or be regulated by ANT (adenine nucleotide translocator), the mitochondrial ADP/ATP nucleotide exchanger, VDAC (the voltage-dependent anion channel), also known as porin, protein cyclophilin D (CypD), the peripheral benzodiazepine receptor, the phosphate carrier, and the FoF1 ATP synthase [38, 39]. CyPD binds to the FoF1 ATP synthase into lipid bilayers to form a Ca^2+^-activated channel that displays features of the mPTP [38]. In this physiological configuration, PTP functions together with other channels and exchangers in the regulation of mitochondrial Ca^2+^ homeostasis [2, 38, 39].

The roles of MOMP and MPT have been mostly considered to participate in the two independent pathways for release of apoptotic and necrotic factors from mitochondria [17]. Bax and Bak interact with the MPT to induce a permeability transition and cytochrome *c* release in isolated mitochondria [40, 41]. Interestingly, intermediate structures common to membrane fusion or fission machineries have also been described to participate in mitochondrial permeabilization phenomenon [17, 42]. New ideas and experiments concerning structures and functions of membrane-permeation channels, exchanger, and pores are under way to confirm whether they act together or separately to promote the mitochondrial events common to apoptotic and necrotic intrinsic pathways [41].

### 3.3. Mitophagy

The outcome of cells with mitochondrial damage can vary. Apoptosis can eliminate ROS-producing cells (cell selection). Autophagy (self-eating) is a catabolic process that targets organelles and cytoplasmic components for degradation by the lysosome [43]. Autophagy has not been considered a modality in cell death; nonetheless, many stimuli that activate apoptosis induce autophagy, whereas signals that inhibit apoptosis inhibit autophagy [43, 44]. The pan caspase inhibitor Z-VAD-fmk inhibits caspases but also blocks lysosomal cathepsins and hence cell death by autophagy. Antiapoptotic proteins, such as Bcl-2 family members, bind to and inhibit beclin (Atg 6), and proapoptotic factors, such as BH3-only proteins, to disrupt this inhibitory interaction and thereby activate autophagy [44]. Autophagy is triggered by ROS derived from either the mitochondrial electron transport chain or NAPDH oxidases. Autophagy of damaged mitochondria limits ROS-modulated caspase-1 activation and seems to negatively regulate pyroptosis [44]. Mitophagy is a specialized form of autophagy in which mitochondria are specifically targeted for degradation at the autophagolysosome, with subsequent degradation by cell's own lysosomal system [44]. Depolarization of mitochondrial membranes is a prerequisite for mitophagy. This process occurs for a period of time for clearance of mitochondria in which damage is not too extensive. Therefore, autophagy of damaged organelles constitutes a survival response that prevents cell death.

## 4. Critical Role of Plasma Membrane Permeabilization by MLKL in the Execution Pathway for Necroptosis 

### 4.1. Necrosome Complex

The interconnected and complex signaling pathways to apoptosis and necroptosis were first recognized by Wallack and Goeddel's groups in 1995-1996. These investigators reported that the interaction of TNF receptors, TRADD and FADD, via DED (death effector domain) was critical for the recruitment of caspase-2, -8, and -10 and the induction of cytotoxicity by TNF family members [45, 46]. The genetic analysis of the caspase-8-deficient and FADD-deficient mice demonstrated the essential role of these genes for embryo development and signaling to tumor necrosis death receptors-induced apoptosis in a physiological setting [45–47]. On the other hand, it was also observed that the cell killing by FADD oligomerization could be caspase-independent suggesting the existence of a particular nonapoptotic cell death program [48–50]. Vercammen and colleagues [51] were the first to report on the induction the morphological features of necroptosis in L929 cells following pharmacological blockage of pan caspase activity by a peptide cell permeable inhibitor named Z-Val-Ala-Asp.fluoromethylketone or ZVAD-fmk.

These studies were confirmed by other authors who identified other cell death genes and small molecule inhibitors that strongly sensitize cells to TNF-*α*, TRAIL, and FasL in eliciting a necrosis-like cell death [48, 52–55]. Degterev and colleagues identified necrostatin-1, a small molecule inhibitor of RIPK1 enzymatic activity [53, 56]. Mitochondria release the second mitochondria-derived activator of caspase (Smac/DIABLO) which bind and inactivate the inhibitors of apoptosis cIAP-1, cIAP-2, and XIAP. Smac mimetics are peptide antagonists of cIAP-1, cIAP-2, and XIAP. The incubation of cells with these peptides enhances TNF-induced necroptosis by promoting autodegradation of cIAP-1, cIAP-2, and XIAP [57] and the formation of RIPK1-dependent complex named necrosome [9].

RIPK1 bears a DD domain allowing its recruitment to TNF-*α*, TRAIL, and CD95 large complexes that initiate the necroptotic cell death pathway [50, 58]. Hitomi and colleagues [54] identified RIPK1 among 7 genes out of 432 genes in a large screening for candidates required for TNF plus zVAD-fmk-induced necroptosis in L929 cells. He and colleagues [55] used a combination of genome-wide siRNA screening and immune precipitation assays to identify RIPK3 as key determinant for necrotic cell death downstream RIPK1. Cho and colleagues [59] undertook a large screening using an siRNA library consisting of 691 human kinase genes to identify additional RIPK1 partners in the induction of necroptosis in FADD-deficient Jurkat cells. RIPK3 was identified as crucial upstream activating kinase that regulates RIPK1-dependent necroptosis* in vitro,* because of its physiological importance in the protection against vaccinia virus infection [59].

Caspase-8 forms with its enzymatically inert homolog cellular FLICE-like inhibitory protein long (cFLIPL) protein, an active complex named ripoptosome. This complex contains caspase-8, FADD, RIPK1, and RIPK3 [13]. Caspase-8 inhibition within this complex blocks the cleavage of RIPK1 and RIPK3 allowing RIPK1 to phosphorylate RIPK3. RIPK3 does not have DD domain in its terminus suggesting other physiological roles in cells [58]. The identification of necrosulfonamide, a small molecule that specifically blocks necroptosis in human cells, was the key to further delineate the downstream pathway mediated by RIPK3 [60]. The mixed lineage kinase domain-like protein (MLKL) was identified as the natural target of RIPK3 kinase [60]. Overexpression of MLKL was able to induce the distinguishable hallmarks of necroptosis [61]. These studies led to the assumption that necroptotic cell death occurs upon the assembly of a large, signal-induced multiprotein complex containing RIPK1, RIPK3, and MLKL which was named necrosome [9, 62]. The RHIM (RIP homotypic interaction motif) domains in molecular structures of RIPK1 and RIPK3 proteins allow the homotypic protein–protein interactions which are essential to form filamentous amyloid structures required for necroptotic signaling [15, 63]. A recent study showed that RIPK3 catalytic activity is dispensable for apoptosis but essential for necroptosis and both apoptosis and necroptosis could proceed simultaneously [64].

### 4.2. MLKL Channel

MLKL is recruited to the necrosome via interaction of its kinase-like domain (KLD) with the kinase domain of RIPK3 [65, 66]. MLKL is a kinase-dead protein. The N-terminal region of MLKL contains a 4HBH (a four-helical bundle domain) with four helices (amino acids 1–125) and, within its N-terminal, two more helices (amino acids 125–181) in its BR domain. MLKL has a pleckstrin homology (PH) domain, similar to the domain of the phospholipase C *δ* (PLC*δ*), pleckstrin, spectrin, and dynamin [67]. This PH domain seems to be required for its insertion into the membrane surface, more specifically, through binding to phosphatidylinositol phosphates [66, 68–70]. The PH domain can bind to different phosphoinositide polyphosphates and inositol polyphosphates [71]. After its phosphorylation by RIPK3, MLKL translocates to lipid rafts inside of the plasma membrane and interacts with the positively charged patch formed by phosphoinositolphosphate (PIPs) molecules. This leads to formation of a high molecular weight pore-forming structure that allows the diffusion of ions, in particular, sodium [68, 69]. This increases intracellular osmotic pressure and eventually leads to membrane rupture [68, 70]. More importantly, MLKL induced-leakage has been demonstrated after it is bound to PIP, PI(4,5)P2, and PI(3,4,5)P3 containing liposomes [69]. All of the above events are not seen in apoptosis. Nonetheless, MLKL may likely function in a similar mechanism proposed to Bax or Bak proteins for membrane insertion and pore formation. Finally, one study has suggested the possible role of MLKL in regulating extracellular Ca^2+^ influx from the transient receptor potential melastatin-related 7, which deserves further investigation [66].

A recent study showed that the RIPK3-MLKL interaction and translocation of necrosomes to mitochondria associated membranes are essential for necroptosis signaling [68]. It was demonstrated that the assembly of RIPK1-RIPK3 complex initiates the intrinsic necroptosis pathway with the participation of PGAM5L and PGAM5S. These two protein phosphatases cause the activation Drp1 (dynamin-related protein 1) and its translocation to the mitochondria. In mammalian cells, mitochondrial fusion is regulated by mitofusin-1 and -2 (MFN-1/2) and optic atrophy 1 (OPA1), whereas mitochondrial fission is controlled by Drp1. Throughout the necrosis process, Drp1 associated with its mitochondrial anchors Fis1 and Mff to induce mitochondrial fragmentation; however, cytochrome *c* is not released as it occurs in the apoptosis intrinsic pathway [72]. Thus both mitochondrial fission and fusion proteins appear to modulate necroptosis through activities that are distinct from their roles in mitochondrial dynamics, a question that remains to be confirmed [73]. However, recent studies have excluded the requirement of PGAM5 and DRP1 in necroptosis [65, 74, 75].

The production of ROS has been shown to be essential for TNF*α*-induced programmed necrosis in L929 cells and mouse embryonic fibroblasts [51]. The importance of the mitochondrial axis in the induction of necroptosis was confirmed in one study showing that once activated RIPK3 kinase initiates the phosphorylation of several downstream target proteins including phospholipase A2 and the proteases calpains and cathepsin [76]. Other critical targets include the cytoplasmatic NOXA1/NADPH oxidase complex and mitochondrial complex I, which are responsible for excessive ROS production, ATP depletion, and opening of the mitochondrial permeability transition pores [76]. These events are accompanied by prolonged JNK activation and stimulation of several metabolic enzymes of glycolysis, glycogenolysis, and glutaminolysis as well as the stimulation of the Krebs cycle [76]. In fact, a previous study has shown that the Nox1 NADPH oxidase has a role in the TNF-induction of necrotic cell death [77].

**Figure 3:**
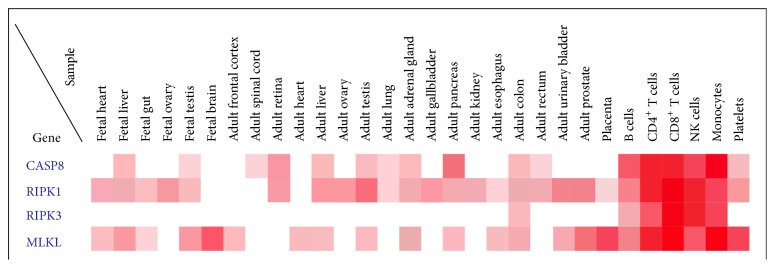
Expression of caspase-8, RIPK1, RIPK3, and MLKL in fetal and adult organs and hematopoietic and myeloid cells. Each row corresponds to each protein concentration displayed as white (no expression) to red color intensity. The heat map was obtained from http://www.humanproteomemap.org/.

Not surprisingly, RIPK1, RIPK3, and MLKL are strongly expressed in immunologic organs like the thymus and spleen and lymphoid and myeloid cell lines, as compared to fetal and adult organs. The relative expression is shown as dendrogram in Figure 3. It is now well established that necroptosis occurs both in T lymphocytes after HIV-1 infection and neutrophils and macrophages after bacterial infection and during tissues injury [9]. A number of pathogens cause host cell death and caspase-8 and RIPK3 are considered the key regulators of macrophage cell death [78, 79]. Future work is needed to clarify the relationship of these metabolic and bioenergetic mechanisms and their overlapping with either early or later plasma membrane associated biochemical events [65, 74, 75].

## 5. Knockout Mice Models for* In Vivo* Cell Death Studies

Mice models have been the key biological tools to define apoptotic and necrotic cell death in development, physiology, and homeostasis as revealed in gene-knockout (KO) mice for a broader number of the TNF and Bcl-2 family members and their regulators [11, 80, 81]. Major phenotypes observed in mice deficient in cell death-pathway genes by intercrossing different null alleles are shown in Table 1. The crossing of these transgenic KO mouse models deficient in two and three genes has helped elucidate cell death necroptotic pathways and the essential role of downstream regulator genes involved in several inflammatory pathologies. Some examples are shown in Table 2.

### 5.1. Mice Models for Lethal Genes

Knockout mice for caspase-8,* caspase-8*−/−, FADD, and* fadd*−/− or double-knockouts for both show an embryonically lethal phenotype due to uncontrolled apoptosis or necroptosis [11, 82, 83]. The embryonic lethality in mice lacking FADD or caspase-8 is due to massive necrosis and can be rescued by RIPK1 or RIPK3 deletion, respectively.* Ripk1*−/− mice die at birth of systemic inflammation and the large area of necrosis in the liver and thymus [84, 85]. On the contrary, knockout of RIPK3 in mice did not cause any measurable defect in development, fertility, NF-*κ*B activation, and apoptosis [86].* Ripk3*−/− mice were resistant to necrotic pancreatitis (cerulein-induced) and vaccinia virus-induced hepatic necrosis [55]. The rescue of a lethal phenotype in RIPK1 knockout is often used as an argument for the implication of necroptosis. Mice deficient in FADD or caspase-8 die during embryogenesis; however, mice with triple deletion of FADD, caspase-8, and RIPK3 are viable [55, 62, 87, 88]. The cardiac, vascular, and hematopoietic defects that occur during the heart development of caspase-8 and FADD knockout mice are caused by RIPK3-mediated necrosis [87, 89]. Therefore, FADD and caspase-8 act as prosurvival factors that suppress the deleterious effects of necrosis by promoting the cleavage and inactivation of RIPK1 and RIPK3. On the other hand, double-knockout mice for* ripk3*−/− and* flip*−/− die during embryonic development due to uncontrolled apoptosis driven by active caspase-8 [89]. These experiments demonstrate that FLIP is an important brake on both apoptotic and necrotic cell death* in vivo.*

Caspase-8 and RIPK3 are all essential for clonal expansion for T and B cell clones [63]. Mice deficient in* Casp8−/−* fail to develop and die* in utero*, which may ultimately be due to failing to maintain the proliferation of T and B cells [90]. After stimulation, T cells lacking caspase-8 or its adaptor protein FADD developed hyperautophagic morphology and die by necroptosis [90]. Proliferation in caspase-8-deficient T cells is fully rescued by crossing with* ripk3−/−* mice, although such rescue ultimately leads to lymphadenopathy [63].

Although TNF-*α* was initially identified by its ability to kill tumor cells, most normal and tumor cells do not undergo cell death in response to this cytokine [91]. The treatment of wild type murine embryonic fibroblasts (MEFs) with TNF does not induce cell death. Overexpression of the* ripk3* gene causes necroptosis in MEFs stimulated with TNF in the absence of RIPK1, caspase-8, Bax, and Bak [92]. RIPK3 activation may be driven by spontaneously high RIPK3 levels or by another RHIM-containing protein such as DAI or TRIF [92]. MLKL is a RHIM-containing protein that is activated in cells with elevated RIPK3 consistent with its important role in necroptosis [92]. RIPK3 can partner also with TLR3 and TLR4 to induce macrophage necrosis and NF-kB activation but this requires TRIF [93].

MLKL is one essential effector of necroptosis since it causes cell death by a caspase- and Bax/Bak-independent mechanism [72]. Mice deficient in* Mlkl *gene are anatomically normal, viable, and fertile [94]. Cells from* Mlkl*−/− mice failed to undergo TNF-mediated necroptosis, as expected. Double deficient mice of* caspase-8* and* Mlkl* genes,* Casp8*−/−;* Mlkl*−/− mice, were normal but showed pronounced splenomegaly, thrombocytopenia, and lymphadenopathy after a few months of age [94]. This phenotype is similar as described by mice lacking functional FAS (*lpr*) or FAS ligand (*gld* mutant mice) [11].

### 5.2. Mice Models for Inflammatory Genes

Various pathological processes such as ischemic brain injury, myocardial infarction, organ transplantation, and virus replication are accompanied by strong inflammatory response. This inflammatory process is characterized by extensive necrosis of tissues. Studies on mouse models with FADD-TNFR1 and FADD-MyD88 deficiency revealed that both TNF and TLR signaling partially contribute to progression of inflammation [55, 88, 95]. Studies on mouse models have also shown that TNF-induced systemic inflammatory response syndrome (SIRS) and CLP-induced peritoneal sepsis are driven by both RIPK1 and RIPK3-dependent necroptosis [12, 58]. The regulated necrosis is also associated with tissue damage and inflammation driven by activation of sterile inflammatory response, including ischemia-reperfusion injury, Alzheimer's disease, atherosclerosis, and toxic insult to liver and lung [9, 96]. A recent study showed that loss of the RIPK3 or MLKL can provide protection to* Ripk1−/−* mice from systemic inflammation but fails to protect these mice from lethal intestinal inflammation [85].* Ripk1−/−* and* Ripk3−/− *mice have significantly lower rates of death and inflammation, whereas the* Ripk1−/−* and* Myd88−/−* mice have reduced inflammation, however, not reduced mortality [85]. Together, these studies demonstrated that only* Ripk1−/−, Ripk3−/−,* and* Casp8−/−* mice are protected from inflammation and intestinal disruption, reach adulthood, and are viable and fertile [83, 85, 89]. Future studies using* Ripk1−/− Ripk3−/−* and* Ripk1−/− Mlkl−/−* mice will be important to understand the role of these proteins in inflammation and the release of cytokines and CDAMPs.

Mice with chronic proliferative dermatitis mutation (cpdm mutant) develop TNF-dependent multiorgan inflammation, which is characterized by dermatitis, liver inflammation, splenomegaly, and loss of Peyer's patches [97]. These mice are deficient in SHARPIN, a protein that, together with HOIL-1, is a key regulator of HOIP (LUBAC), an ubiquitin ligase complex that catalyzes the addition of linear ubiquitin to target proteins [15]. SHARPIN is required for the TNFR signaling complex and for prevention of cell death in various cells, including epidermal keratinocytes [15]. A recent study has demonstrated that RIPK3 or MLKL deficiency is able to prevent liver inflammation in* shpn−/−* mice as well as restoring splenic architecture splenic phenotype and leukocytosis [97]. Only combined caspase 8 heterozygosity and RIPK3 deficiencies were able to almost completely prevent chronic proliferative dermatitis in* shpn−/−* mice. The role of SHARPIN in the skin-inflammation phenotype is mainly due to keratinocyte mitochondria-dependent apoptosis [85]. Since RIPK3 and MLKL deletion markedly reduced leukocytosis, it is suggested that the hematopoietic phenotype is mediated by necroptosis [97].

The E3 ligases, cIAP-1 (*Clap 1 gene*), cIAP-2 (*Clap 2 gene*), and Xiap, are responsible for RIPK1 polyubiquitination, but knockout mice of these genes are surprisingly phenotypically normal. Double-knockout* Xiap−/− Clap2−/−* mice are also phenotypically normal. However,* Xiap−/− Clap1−/−* and* Clap1−/− Clap2−/−* double-knockout mice are embryonic lethal and this can be rescued by crossing these animals with* Ripk1−/−* and* Ripk3−/−* mice [98].

Mice deficient in Cylindromatosis (CYLD) gene are viable and fertile during adulthood life. CYLD deficiency leads to hyperubiquitinated RIPK1 in the necrosome and impaired phosphorylation of RIPK1 and RIPK3, thereby blocking caspase-8 activation. CYLD is a caspase-8 substrate and when activated it cleaves CYLD and prevents necroptosis, similar to what occurs with the cleavage of RIPK1 and RIPK3 [99]. CYLD is required for TLR3 or TLR4 receptor-induced necroptosis. The crossing of* cyld−/−* mice with other members of the NLR and TLR families promises to deliver further experimental evidence on its crucial role in innate response. Finally, deficiency of both RIPK3 and caspase-8 or FADD completely abrogated Yersinia-induced cell death and caspase-1 activation. Mice ablated of RIPK3 and caspase-8 genes in their hematopoietic compartment showed high susceptibility to Yersinia infection as well as displayed a very low production of proinflammatory cytokines by monocyte and neutrophils [100]. We are waiting for the participation of additional components in these pathways and more complex interactions among them in the intracellular resolution of infections.

### 5.3. Mice Models for Mitochondria-Associated Channel and Pore Genes

Ischemia and reperfusion injury (IRI) cause a wide array of functional and structural alterations of mitochondria. Ca^2+^ Overload and MPTP openings are critical steps in this process. MPT is critically dependent on ANT, the ADP/ATP nucleotide exchanger, and the mitochondrial protein cyclophilin D (CypD), the regulator of ANT. Studies have been done to prove the parallel existence of two independent pathways of regulated necrosis in ischemia-reperfusion injury [101, 102]. Knockout of the gene encoding CypD renders mitochondria resistant to Ca^2+^ overload-induced swelling and the heart and brain partially resistant to cell death due to ischemic injury [103]. RIPK3-deficient mice are protected from IRI as well as from hyperacute TNF*α*-induced shock [104]. The* in vivo* analysis of cisplatin-induced acute kidney injury and hyperacute TNF-shock in Cyp-D and RIPK3 double-deficient mice demonstrated striking differences, but animals died after no longer than 120 h [102]. Protection of RIPK3-knockout mice was significantly stronger than for CypD-deficient mice. Cyclosporin prevents CyP-D binding to the ANT, whereas sanglifehrin (SfA) inhibits the peptidyl-prolyl cis-transisomerase (PPIase) activity of CyP-D but does not prevent its binding to the ANT. The protection from kidney injury upon long ischemia was observed when applying a combination of Nec-1 (necrostatin) and SfA, but the protection was not complete [102]. Necrostatin-1 blocks necroptosis in many settings by interacting and inhibiting RIPK1 kinase activity, which is required for activation of RIPK3. Nonetheless, necrostatin failed to block necroptosis in the absence of RIPK1, suggesting the RIPK1 has a protective role [104]. Moreover, mice with a kinase-dead mutation of RIPK1 show normal development and maturation, although cells from these animals appear to be defective in TNF-induced necroptosis [62].

The* Bax*−/−*Bak*−/− mice typically die at a perinatal age with multiple developmental defects, and only 10% survive into adulthood [81]. Mouse embryonic fibroblasts (MEFs) from* Bax*−/−* Bak1*−/− double-knockout (DKO) grown in culture are highly resistant to cell death by the apoptotic inducer staurosporine, confirming their critical role in apoptosis [105]. Recently, Karch and colleagues demonstrated that these cells are also resistant to H_2_O_2_, ionomycin, and DNA alkylation agents, which are inducers of necrotic phenotypes [106]. Consistent with results in DKO MEFs, cardiac-specific deletion of Bax/Bak1 significantly protected the heart from ischemia-reperfusion (I-R) injury and reduced mortality in mice subjected to permanent myocardial infarction injury [106]. MEFs from* Ppif* (CypD) deficient mice are insensitive to Ca^2+^-induced MPTP opening, and Bax/Bak does not directly affect the MPTP at the level of the inner membrane. However, the authors concluded that, in the absence of BAX/BAK, the outer membrane resists swelling and prevents organelle rupture, thereby preventing cell death. The antiapoptotic family members, Bcl-2 and Bcl-xL, act directly or indirectly to preserve the integrity of the outer mitochondrial membrane. To examine this issue, the authors showed that the Bcl-2/Bcl-xL inhibitor ABT-737 sensitized the MPTP to open with mild Ca^2+^ stimulation or ionomycin treatment in wild type but not in Bax/Bak1 DKO cells. In light on these results, the authors concluded that Bax/Bak oligomers within the outer membrane are required for mitochondrial permeability pore-dependent cell death by serving as a necessary functional component of the MPTP [106]. Similar observations were previously reported by Irrinki and colleagues [107]. These authors investigated necroptosis induced by TNF, cycloheximide (CHX) plus z-VAD-fmk in MEFs derived from mice bearing two or three knockout genes including* fadd*−/−,* rip1*−/−,* NEMO*−/−,* caspase-8*−/−,* bad−/−bak−/−* (double-knockout), and* bax*−/−*bak*−/− (double-knockout) genes. Moreover, it was demonstrated that overexpression of Bcl-xL protects cells from necroptosis induced by these agents [107]. Other putative mechanisms through which the outer membrane proteins could affect formation of the MPTP are reviewed in [38].

Future work along these lines using RIPK-3 and MLKL-deficient mice as well as the combination with other mutant mice may uncover the complex regulation and interconnectivity among apoptosis effectors of mitochondrial intrinsic pathway and regulated necrosis execution pathway.

## 6. Conclusion and Future Directions

In the last decade, a number of cellular and molecular studies have advanced the knowledge and acceptance that in response to a variety of stress conditions, physicochemical insults, viral and bacterial infection, necroptosis rather than apoptosis emerges as predominately cell death in tissues into adult life.

We have highlighted the intricately connected and overlapping extrinsic and intrinsic molecular pathways regulating both apoptosis and necroptosis. We now know that besides TNF family of receptors many other cell-surface and intracellular receptors, including TLRs, NLRs, IFNRs, and T-cell receptors, can induce regulated cell death. Similarly, intracellular deregulation and/or imbalance of synthesis and metabolic pathways inside mitochondria, endoplasmic reticulum, and intracellular organelles can provoke regulated cell death. Exploring and classifying the molecular mechanisms underlying the initiation and execution pathways will help us search for new strategies to enhance or inhibit necroptotic cell death rates in pathological clinical scenarios such as inflammation, cancer resistance to chemotherapy, and excessive tissue death as result of ischemia and injury.

Caspase-8 and cFLIP heterodimers act as key regulators of death checkpoint to alternate the cell death pathway and canonic and noncanonical NF-*κ*B signaling. Intracellular downstream signaling components of the TNFR multicomplexes and NF-*κ*B signaling complexes, such as E3 ligases, cIAP-1, and cIAP-2, deubiquitinases, CYLD, A20, and HOIL-1/HOIP/Sharpin (LUBAC), are equally very important positive and negative regulators of inflammation and cell death decision checkpoints to apoptosis and necroptosis.

Mice models lacking the caspase-8, FADD, or FLIP are embryonic lethal and are rescued with ablation of RIPK1 and RIPK3 genes. With the help of gene-deficient mice models we will advance in the studies aiming to further understand the precise roles of these proteins at cell decision points from apoptosis to necroptosis and inflammation. TNF can activate RIPK3 in the absence of RIPK1 and surprisingly RIPK3 has a proinflammatory role itself. Future studies need to be done to understand how RIPK3-dependent necroptotic cell death can be activated in an RIPK1-independent manner. In this regard, the RHIM-containing protein such as DAI and TRIF as well as IFNAR merits further investigation because of its role in virus-induced necroptosis. Many other landmark genes will have place in the molecular pathways leading to regulated cell death. Knockout mice and their cell lines lacking essential genes will be important to investigate cross talk and kinetics aspects of cell death triggering, propagation, and resolution. These animal models will have critical role to future preclinical tests of new drugs for inhibiting cell death causing human diseases.

The plasma membrane permeabilization induced by MLKL oligomers plays a key role in the execution pathway for necroptosis. The 4HBH phospholipid-binding domain acts by recruiting the MLKL protein to bind to lipid ligands embedded in the membrane surface. Since PIP, PI(4,5)P_2_, and PI(3,4,5)P_3_ are abundant phospholipids in ubiquitous cell membranes, it is crucial to establish whether MLKL binding and pore-forming molecular mechanism can be specifically and efficiently modulated by combination of membrane lipids. Elucidating the combinatorial “codes” and critical domain structure for this interaction will help us design and develop new therapeutic strategies to prevent cell death triggered in prolonged degenerative, inflammatory, and infectious diseases.

## Figures and Tables

**Table 1:** Summary of phenotypes of double- and triple-knockout mice ablated for genes that regulate apoptotic and necroptotic cell death pathways.

Crossed mice	*ripk1−/−* die postnatal days 1-2	*ripk3−/−* viable	*mlkl−/−* viable	*ripk1−/−* *ripk3−/−* die postnatal day 4	*ripk1−/−* *mlkl−/−* die postnatal day 4
*casp8−/−* **Embryonic lethal**	**Embryonic lethal**	Viable	**Embryonic lethal**	Viable fertile	Die postnatal day 4

*flip−/−* **Embryonic lethal**	**Embryonic lethal**	**Embryonic lethal**	**Embryonic lethal**	**?**	**?**

*fadd−/−* **Embryonic lethal**	**Embryonic lethal**	Viable lympha-denopathy	**Embryonic** **lethal**	Viable fertile	Viable fertile

*casp8−/−* *fadd−/−* **Embryonic lethal**	**Embryonic lethal**	Viable	**Embryonic lethal**	Viable fertile	**?**

*flip−/−* *fadd−/−* **Embryonic lethal**	**Embryonic lethal**	Viable lympha-denopathy splenomegaly	**Embryonic lethal**	Viable fertile	**?**

*bax−/−* *bak−/−* viable (10%)	**Embryonic lethal**	**?**	**?**	**?**	**?**

*cypd−/−* viable	**Embryonic lethal**	Viable partial protection of cardiac ischemia	Viable	**?**	**?**

Summary of knockout mice models of apoptosis and necroptosis genes for which the phenotypes reveal a critical role in development, physiology, and homeostasis. Caspase-8, FLIP, and FADD proteins have pivotal roles in the death inducing signaling complex that regulate apoptosis FLIP deficiency causes both massive apoptosis and necrosis. Knockout mice for caspase-8, *Casp-8*−/−, FADD, and *Fadd*−/− or double knockouts for both show an embryonically lethal phenotype due to uncontrolled necrosis. Knockout mice for RIPK1, *Ripk1*−/−, die at birth of systemic inflammation whereas *Ripk3*−/− mice are normal but are resistant to proinflammatory stimuli. *Mlkl*−/− mice are anatomically normal, viable, and fertile. Triple knockouts mice *Fadd*−/− *Flip*−/− *Ripk3*−/− have a normal cell-death pathway and develop to normal birth because of absence of necrosis and apoptosis which are modulated by caspase-8. *Casp8*−/− *Mlkl*−/− double knockout mice are normal and resistant to TNF-induced necroptosis. *Bax−*/*− Bak−*/*−* double knockout mice develop perinatal lethality and only 10% survive into adulthood, and these mice develop splenomegaly and lymphadenopathy. The question mark indicates possible or still unknown.

**Table 2:** Summary of phenotypes in double- and triple-knockout mice ablated for genes that regulate apoptosis, necroptosis, and inflammation.

Crossed mice	*ripk1−/−* die postnatal day 1	*ripk1−/−* *tnfr−/−* die postnatal day 1	*sharpin−/−* viable die postnatal days 10–14 inflammation	*a20−/−* viable die postnatal days 7–14 inflammation	*cyld−/−* viable reach adulthood inflammation
*ripk3−/−* *casp8−/−* viable	Viable	Viable?	**Embryonic** **lethal**	**Embryonic** **lethal**	?

*ripk3−/−* *fadd−/−* viable	Viable	Viable?	**Embryonic** **lethal**	**Embryonic** **lethal**	?

*ripk3−/−* *tnfr−/−* viable	Reach adulthood	Viable?	Prevent inflammation skin dermatitis	Prevent inflammation Cachexia	?

*ifnar−/−* viable	Die postnatal day 1	Die later than *ripk1−/−* *tnfr1−/−*	?	?	?

*trif−/−* viable	Die postnatal day 1	Die later than *ripk1−/−* *tnfr1−/−*	?	?	?

*ripk3−/−* *mlkl−/−* die postnatal day 4	Die postnatal day 4	Die later than *ripk1−/−* *tnfr1−/−*	Prevent systemic inflammation	?	?

Summary of phenotypes for double- and triple-knockout mice models of apoptosis, necroptosis, and inflammation genes. Knockout mice for RIPK1, *Ripk1*−/−, die at birth of systemic inflammation whereas *Ripk3*−/− mice are normal but are resistant to proinflammatory stimuli. *Mlkl*−/− mice are anatomically normal, viable, and fertile. Triple-knockout mice *Fadd*−/− *Flip*−/− *Ripk3*−/− have a normal cell-death pathway and develop to normal birth because of absence of necrosis and apoptosis which are modulated by caspase-8. *Casp8*−/− *Mlkl*−/− double knockout mice are normal and resistant to TNF-induced necroptosis. Deletion of *Tnfr* gene provides protection from *Ripk1*−/− perinatal lethality and double KO mice *Ripk1*−/− *tnfr−/−* can be partially protected from lethality from systemic inflammation by mating these mice with *ifnar*−/− or *trif*−/− mice. This indicated that both proteins can engage RIPK3-MLKL interaction independent of RIPK1. A20 and CYLD target similar molecular substrates including TRAF2, TRAF6, NF-*κ*B essential modulator (NEMO), and RIPK1. The deubiquitinase CYLD removes the K63-Ub of RIPK1, and A20 promotes the removal of K63-linked ubiquitin chains to terminate signaling induced NF-*κ*B activation. The ablation of A20 and SHARPIN genes is potentially lethal. A20/TNFAIP3, zinc finger and ubiquitin editing protein, CYLD, cylindromatosis, deubiquitylating enzyme; SHARPIN, a protein that together with HOIL-1 and HOIP forms the LUBAC, the heterotrimeric linear ubiquitin chain assembly complex; both are involved in the TNF signaling pathways; IFNAR, the type I IFN receptor. The question mark indicates possible or still unknown phenotype.
